# Nociceptors in cardiovascular functions: complex interplay as a result of cyclooxygenase inhibition

**DOI:** 10.1186/1744-8069-2-26

**Published:** 2006-08-17

**Authors:** Louis S Premkumar, Manish Raisinghani

**Affiliations:** 1 Department of Pharmacology, Southern Illinois University School of Medicine Springfield, IL 62702, USA

## Abstract

Prostaglandins (PGs) are requisite components of inflammatory pain as indicated by the efficacy of cyclooxygenase 1/2 (COX1/2) inhibitors. PGs do not activate nociceptive ion channels directly, but sensitize them by downstream mechanisms linked to G-protein coupled receptors. Antiinflammatory effects are purported to arise from inhibition of synthesis and/or release of proinflammatory agents. Release of these agents from peripheral and central terminals of sensory neurons modulates nociceptive input from the periphery and synaptic transmission at the first sensory synapse, respectively. Heart and blood vessels are densely innervated by sensory nerve endings that express chemo-, mechano-, and thermo-sensitive receptors. Activation of these receptors mediates synthesis and/or release of vasoactive agents by virtue of their Ca^2+^permeability. In this article, we discuss that inhibition of COX2 reduces PG synthesis and renders beneficial effects by preventing sensitization of nociceptors, but at the same time, it might contribute to deleterious cardiovascular effects by compromising the synthesis and/or release of vasoactive agents.

## Synthesis and functions of arachidonic acid and its metabolites

Arachidonic acid (AA) and its metabolites are involved in several important cardiovascular functions. In this article, we address the adverse cardiovascular effects that arise as a result of block of PG mediated modulation of nociceptive ion channels. AA is produced from membrane phospholipids by phospholipase A_2 _(PLA_2_), a calcium-dependent enzyme, which is activated by proinflammatory agents and shear stress exerted on the vessel wall. Activation of phospholipase C (PLC) hydrolyzes phosphatidyl inositol 4, 5 bisphosphate (PIP_2_) to inositol 1, 4, 5 trisphosphate (IP_3_) and diacyl glycerol (DAG). DAG activates protein kinase C (PKC) and DAG lipase, activation of DAG lipase can in turn produce AA. Activation of phospholipase D produces anandamide, which can subsequently be converted to AA by fatty acid amide hydrolase [1].

AA is metabolized via cyclooxygenase (COX1/2), lipoxygenase (5, 12, 15, LOX) and cytochrome P450 (CYP) pathways. COX1 is constitutively active, whereas COX2 is inducible, except in the kidneys and in some parts of central nervous system, where it is expressed constitutively [2]. Cyclooxygenase activation produces prostaglandin H_2 _(PGH_2_), which is subsequently metabolized to PGD_2_, PGE_2_, PGF_2α_, PGI_2 _and thromboxane A_2 _(TxA_2_) [1].

**Figure 1:**
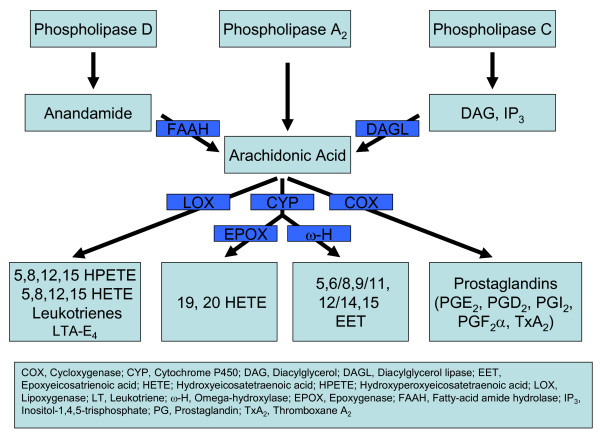
Schematic diagram showing the pathways involved in synthesis and metabolism of AA.

Initial lipoxygenase products 5, 8, 12 and 15-(S) hydroperoxyeicosatetraenoic acids (HPETEs) are subsequently metabolized to 5, 8, 12, 15-(S) hydroxyeicosatetraenoic acids (HETEs). 5-HETE is metabolized to leukotriene A_4 _(LTA_4_), which can be converted to other leukotrienes (LTB_4_-E_4_). LTA_4 _can also be converted to lipoxins by 12- and 15-LOX. AA can also undergo ω-hydroxylation by several isoforms of CYP enzymes leading to the production of 19- and 20-HETE. Several families of CYP also convert AA into epoxyeicosatrienoic acids (EETs) [1] (Fig. 1). The distribution, coupling mechanisms and actions of AA metabolites on cardiovascular system are shown in Table 1.

**Table 1:** Cardiovascular functions of AA and its metabolites

AA Metabolite	Receptor subtypes	Secondary messenger mechanisms	Tissue distribution of the receptors	Cardiovascular functions of AA metabolites	Ref.
PGD_2_	DP1, DP2 (CRTH_2_)	Gs (DP1, 2), Gi, Gq, MAPK (DP2)	Leptomeninges, Langerhan cells, Goblet and columnar cells in GI tract, Eosinophils for DP1, All tissues for DP2	Vasodilation, Vasoconstriction, Platelet deaggregation	1, 12
PGE_2_	EP1, EP3, EP3, EP4	Gs, Gi, Gq	Kidney, Lung and Stomach for EP1, EP2 expressed in response to LPS and gonadotrophins, EP3 and 4 in all tissues	Vasodilation, Vasoconstriction, Maintain renal blood flow and GFR, Vascular smooth muscle mitogenesis	1, 12, 15
PGI_2_	IP	Gs (predominant), Gi, Gq	Neurons, (primarily DRGs), Endothelial cells, Vascular smooth muscle cells, Kidney, Thymus, Spleen and Megakaryocytes	Vasodilation, Inhibit platelet aggregation, Inhibit TXA_2_-induced vascular proliferation	1, 12, 21, 58
PGF_2α_	FP	Gq, EGFR	Corpus luteum, Kidney, Heart, Lung and Stomach	Vasoconstriction, Mitogenesis in heart, Inflammatory tachycardia, Renal functions	1, 12
TXA_2_	TP	Gq, Gs, Gi, Gh, G12	Kidney, Heart, Lungs, Platelets and Immune cells	Platelet aggregation, Vasoconstriction, Inflammatory tachycardia	1, 12, 58
20-HETE	?	Gq, Tyrosine kinase, Increased conductance of L-type Ca^2+ ^channels, Inhibition of Na+-K+-2Cl cotransporter	?	Renal and cerebral artery contraction, Antagonize EDHF mediated vasorelaxation, Myogenic constriction, Regulate renal functions	1, 54
Leukotrienes (LTB_4_-E_4_)	BLT1, BLT2 (LTB_4_), CysLT_1_, CysLT_2 _(LTC_4_-D_4_)	?Gi/Go (BLT1,2, CysLT_1,2_), Gα_16 _(BLT1,2)	Leukocytes, spleen, thymus, bone marrow, lymph nodes, heart, skeletal muscle, brain and liver for BLT1, Most tissues for BLT2,	Coronary smooth muscle contraction, Transient pulmonary and systemic hypertension	1, 54
EETs	?	Gs, Tyrosine kinases, ERK1/2, p38 MAPK, Activation of Ca^2+^-activated K^+ ^channels	?	Renal and cerebral vasodilation, Renal vasoconstriction, Vascular smooth muscle and endothelial cell proliferation	1

## Role of sensory innervation in the cardiovascular system

Noxious stimuli are transduced by peripheral nociceptors, which transmit nociceptive information to pain processing centers in the brain via the spinal cord. Heart and blood vessels are densely innervated by sensory nerve endings that express chemo-, mechano-, and thermo-sensitive receptors, which include acid sensitive ion channels (ASIC), degenerin/epithelial sodium channels (DEG/ENAC), purinergic ATP gated ion channels (P2X), and transient receptor potential (TRP) channels [3-7]. Activation of nociceptive ion channels, particularly ASIC3 and TRPV1, has been implicated in ischemic cardiac pain [5]. Both these channels can be activated by acidic pH and sensitized by proinflammatory agents synthesized and/or released during ischemia.

Activation of Ca^2+ ^permeant nociceptive ion channels on the peripheral and central terminals of sensory neurons leads to the synthesis and/or release of a variety of proinflammatory agents and neuropeptides, like bradykinin (BK), PGs, calcitonin gene-related peptide (CGRP), substance P (SP), vasoactive intestinal peptide (VIP) and adenosine triphosphate (ATP) etc. [8,9]. Increases in intracellular Ca^2+ ^initiate several second messenger pathways, including activation of PLA_2_, PLC and Ca^2+^-dependent kinases, which can lead to the generation of AA and its metabolites, release of Ca^2+ ^from intracellular stores, and phosphorylation of nociceptive receptors, respectively. BK is thought to be synthesized and released on demand from sympathetic nerve endings [11]. BK initiates prostanoid synthesis and mediates release of vasoactive neuropeptides [10,11]. PGE_2 _and PGI_2 _are produced in response to nociceptive stimuli and lead to inflammation and pain by sensitization of nociceptors. PGI_2 _is a potent vasodilator and platelet deaggregator [12]. In blood vessels, activation of nociceptive receptors results in an endothelium independent vasodilatory response, which is mediated mainly by the release of CGRP [13]. CGRP is a potent vasodilator (coronary vasculature is particularly sensitive) that increases both heart rate and contractile force [13,14]. SP and VIP released from sensory nerve terminals induce vasodilation and positive chronotropic effect [15]. ATP is released ubiquitously along with neurotransmitters and induces vasoconstriction by activation of P2X receptors, however, its breakdown product adenosine is a potent vasodilator and also inhibits neurotransmitter/neuropeptide release [16]. Relatively less prominent vasoactive agents are also released from the nociceptive nerve endings including galanin, corticotrophin-releasing factor, arginine, cholecystokinin-octapeptide, neuropeptide K, eledoisin-like peptide and bombesin-like peptides [14]. Nociceptor stimulation not only serves as a sensory-afferent, but also plays a significant role in sensory-efferent functions [8]. It has also been postulated that vascular regulation via an efferent mechanism could be independent of the sensory afferent function [17] and the selective synthesis and/or release of specific vasoactive agents could arise from the nature of the stimulus and/or its intensity [18]. Thus, activation of Ca^2+ ^permeable nociceptive ion channels at the peripheral and central terminals of sensory neurons can play an important role in the synthesis and/or release of vasoactive agents.

## Nociceptive ion channels in cardiovascular system

Several nociceptive ion channels have been cloned. Most of these channels are modulated by PKA and PKC mediated phosphorylation. Significantly, PGE_2 _and PGI_2 _mediate their effects by activation of PKA and PKC pathways. The Transient Receptor Potential (TRP) channels (TRPVanilloid, TRPAnkyrin, TRPClassical, and TRPMelastatin) are chemo-, mechano-, and thermo-sensitive. TRPV1 is a well-characterized channel, which transduces heat in the noxious temperature range (>42°C) and is critical for inflammatory thermal sensation [19]. It is a Ca^2+ ^permeant polymodal receptor activated by protons, anandamide, lipoxygenase metabolites of AA, N-arachidonyl dopamine, capsaicin (an active ingredient in hot chilli peppers) and resiniferatoxin (RTX, an ultrapotent agonist obtained from the cactus, *Euphorbia resinifera*) [20]. TRPV1 is distributed in the heart and blood vessels and is sensitized by PGs via PKA and PKC mediated phosphorylation [21]. Importantly, in the phosphorylated state, the activation threshold of TRPV1 is reduced below body temperature rendering the channel constitutively active [20]. Furthermore, phosphorylation also promotes translocation of TRPV1 from the cytosol to the plasma membrane [22,23]. Activation of TRPV1 in sensory nerve endings supplying heart and blood vessels releases multiple vasoactive agents [14]. In diabetes, TRPV1 has been shown to be downregulated, which might contribute to the cardiovascular complications [23].

The role of TRPV1 in the cardiovascular system has been addressed: 1) Infusion of TRPV1 agonists significantly alters blood pressure, which could be mostly reversed by selective TRPV1 antagonists [24,25]; 2) Ablation of TRPV1 expressing C fiber terminals by capsaicin or resiniferatoxin (RTX) results in the loss of CGRP release, increased plasma renin activity, and an inability to control salt loading by the kidneys [14]; 3) Activation of TRPV1 or ASIC3 by protons during ischemia mediates a sympathoexcitatory reflex that is abolished by RTX treatment [5,26].

Inhibition of COX leads to increased metabolism of AA via LOX and CYP pathways. Products of LOX pathway (12- and 15-(S)-HPETE, 5- and 15-(S)-HETEs and LB_4_) can directly gate TRPV1 [20]. Myogenic constriction in response to increased pressure on the intraluminal surface of blood vessels is mediated by the CYP byproduct 20-HETE, which directly activates TRPV1 and releases SP [27].

We propose that reduction of PG levels may contribute to deleterious vascular effects by decreasing sensitization of TRPV1 and subsequent reduction of CGRP and SP release. This possibility is supported by the finding that recovery from myocardial ischemia is compromised in TRPV1 knockout mice [28] and proton mediated CGRP release from the heart is mediated exclusively by TRPV1 [29,30]. Since TRPV1 antagonists may become a part of the therapeutic armamentarium for painful conditions [31], it is imperative to determine if blocking nociceptive receptors like TRPV1 decreases the release of vasoactive agents that are essential for homeostasis of the cardiovascular system.

TRPV2 is 50% identical to TRPV1 and mediates high-threshold (>52°C) noxious heat sensation. In arterial myocytes, TRPV2 is activated by stretch, which is an important stimulus in cardiovascular functions [32]. Cardiac-specific transgene expression of TRPV2 results in Ca^2+^-overload-induced cardiomyopathy [32]. TRPV3 is activated by temperatures >31°C and is involved in nociception [32]. TRPV4 is activated by temperatures >25°C and its activity is augmented by hypotonicity. PGE_2 _potentiates TRPV4 and exacerbates pain behavior in animals, whereas EET directly activates the channel [32,33]. TRPV4 is found abundantly on endothelial and vascular smooth muscle cells of intralobar pulmonary artery and aorta where, it mediates calcium influx [34]. TRPM8 is a Ca^2+ ^permeant innocuous cold temperature sensor, which plays a role in nociception [36] and mediates Ca^2+ ^influx into vascular smooth muscle cells [34].

Mechanosensitive channels play a major role in cardiovascular functions and the identity of these channels is becoming apparent with cloning of TRPC1 and TRPA1 [32]. TRPC1, 2, 3, 4 and 6 are present on endothelial cells, activation of which increases intracellular Ca^2+ ^[35]. DEG/ENAC belongs to a family of mechanosensitive channels, which include ASICs and their splice variants [37]. ASICs are modulated by AA, PKC and PKA [37-41]. ASIC1 behaves as a mechanosensor only in viscera, but not in the periphery [42,43]. Activation of ASIC3 has been postulated to carry ischemic cardiac pain [5].

Chemo-sensitive purinergic receptors (P2X_1–6_) are activated by extracellular ATP. The P2X_3 _receptor subtype is expressed exclusively in small and medium diameter dorsal root and trigeminal ganglia neurons [44]. In the cardiovascular system, activation of P2X_4 _receptor increases cardiac contractility [45]. Activation of P2X mediates AA production via stimulation of PLA_2 _[46]. P2X_1,2,7 _channels are also regulated by PKC [47-49]. P2X_1 _is present on vascular smooth muscle cells and mediates vasoconstriction by ATP released from sympathetic nerve activity [50].

**Figure 2:**
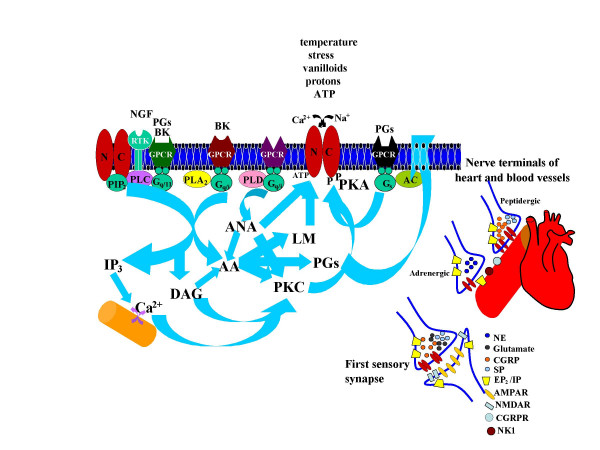
Second messenger pathways that modulate nociceptive ion channels.

From these studies it is clear that several nociceptive ion channels are modulated by activation of PKA and PKC, therefore, it is reasonable to expect that PGs coupled to these pathways would be able to sensitize the nociceptive ion channels. Thus, in our opinion, it is highly probable that the block of PG synthesis by COX inhibitors affects the cardiovascular functions mediated by nociceptive ion channels (Fig. 2).

## Advantages and disadvantages of selective inhibition of COX2

Although COX2 inhibitors have become popular, their analgesic effects are comparable to non-specific COX inhibitors [51]. The selectivity of COX2 inhibitors has a significant advantage of avoiding gastrointestinal side effects (VIGOR study) due to the preservation of PGE_2 _levels and a reduction in the incidence of colon cancer by inhibition of PG-mediated angiogenesis [52-54]. The inducible nature of COX2 is claimed to have significant advantages because it is activated only at the sites of inflammation. In this regard, it is significant to note that atherosclerotic lesions are inflammatory in nature [55] and PGI_2 _(vasodilator, platelet deaggregator and sensitizer of nociceptive receptors) is synthesized via COX2 activation as a necessary protective mechanism. Nonspecific COX inhibitors decrease production of both, PGI_2 _and TxA_2 _(platelet aggregator), thereby avoiding an imbalance between PGI_2 _and TxA_2 _levels [56]. In contrast, when COX2 is inhibited selectively, platelet aggregation by TxA_2 _is intact, but at the same time PGI_2 _induced platelet deaggregation is compromised, resulting in enhanced platelet aggregation [57]. Here, we propose that inhibition of PGE_2 _and PGI_2 _could also reduce sensitization of nociceptors and compromise release of potent vasodilators in response to ischemia, which could be critical in reversing hypoperfusion in conditions like myocardial ischemia. Indeed, injury-induced platelet activation is enhanced in PGI_2 _receptor (IP) knock-out mice [58], whereas it is reduced in TxA_2 _receptor (TP) knock-out mice [58]. These findings are consistent with patients treated with COX2 inhibitors suffering from higher incidence of MI and stroke as compared to naproxen treated patients [53,59,60]. A combination of a COX2 and a low dose of COX1 inhibitors (for example, 80 mgs of aspirin) may be a beneficial strategy to prevent TxA_2_-mediated platelet aggregation. Furthermore, the need for platelet deaggregation becomes even more critical, given the lifetime risk of developing atrial fibrillation significantly increases over 40 years of age [61], which can initiate thromboembolism.

## Concluding remarks and future directions

The beneficial effects of COX inhibitors are derived from their ability to inhibit synthesis of PGs. However, several important cardiovascular functions mediated by PGs are compromised, including direct vasodilation, vasoconstriction, and platelet aggregation/deaggregation. Herein, we propose that the ability of PGs to sensitize nociceptive ion channels involved in the release of potent vasoactive agents could also be compromised. A well-characterized receptor in this context is TRPV1, which is sensitized by PGs and its activation mediates the synthesis and/or release of vasoactive agents by virtue of its high Ca^2+ ^permeability. TRPV1 is currently being pursued as a potential target for the next generation of analgesics [31]. Use of COX inhibitors should be dictated objectively by understanding the mechanisms by which cardiovascular complications are induced, instead of being swayed by emotional testimonies in congressional inquires. Drug industries would be better advised to invest in research rather than spending billions (3 billion in 2004) in advertising and direct marketing to patients. Judicious use of these drugs with open dialogue between drug industries, physicians and patients must be encouraged, so that all the parties involved can make an informed decision, fully aware of the consequences. Patients who are in the right category would benefit from these drugs, while sparing others who are at a risk for cardiovascular complications. This strategy/approach will also avoid expensive class action lawsuits and prevent driving the cost of medication higher; otherwise, patients who need the medication most may not be able to afford.
